# Novel autosomal dominant *TMC1* variants linked to hearing loss: insight into protein-lipid interactions

**DOI:** 10.1186/s12920-023-01766-7

**Published:** 2023-12-08

**Authors:** Sung Ho Cho, Yejin Yun, Dae Hee Lee, Joo Hyun Cha, So Min Lee, Jehyun Lee, Myung Hwan Suh, Jun Ho Lee, Seung-Ha Oh, Moo Kyun Park, Sang-Yeon Lee

**Affiliations:** 1 Seoul National University College of Medicine, Seoul, South Korea; 2 Department of Otorhinolaryngology-Head and Neck Surgery, Seoul National University Hospital, Seoul National University College of Medicine, Jongno-Gu, Daehak-Ro, 101, Seoul, South Korea; 3 CTCELLS, Inc, 21, Yuseong-daero, 1205beon-gil, Yuseong-gu, Daejeon, Republic of Korea; 4 Sensory Organ Research Institute, Seoul National University Medical Research Center, Seoul, South Korea; 5 Department of Genomic Medicine, Precision Medicine & Rare Disease Center, Seoul, South Korea

**Keywords:** *TMC1*, Hearing loss, Structural modeling, DFNA36, Protein-lipid interaction

## Abstract

**Background:**

*TMC1*, which encodes transmembrane channel-like protein 1, forms the mechanoelectrical transduction (MET) channel in auditory hair cells, necessary for auditory function. *TMC1* variants are known to cause autosomal dominant (DFNA36) and autosomal recessive (DFNB7/11) non-syndromic hearing loss, but only a handful of *TMC1* variants underlying DFNA36 have been reported, hampering analysis of genotype-phenotype correlations.

**Methods:**

In this study, we retrospectively reviewed 338 probands in an in-house database of genetic hearing loss, evaluating the clinical phenotypes and genotypes of novel *TMC1* variants associated with DFNA36. To analyze the structural impact of these variants, we generated two structural models of human TMC1, utilizing the Cryo-EM structure of *C. elegans* TMC1 as a template and AlphaFold protein structure database. Specifically, the lipid bilayer-embedded protein database was used to construct membrane-embedded models of TMC1. We then examined the effect of *TMC1* variants on intramolecular interactions and predicted their potential pathogenicity.

**Results:**

We identified two novel *TMC1* variants related to DFNA36 (c.1256T > C:p.Phe419Ser and c.1444T > C:p.Trp482Arg). The affected subjects had bilateral, moderate, late-onset, progressive sensorineural hearing loss with a down-sloping configuration. The Phe419 residue located in the transmembrane domain 4 of TMC1 faces outward towards the channel pore and is in close proximity to the hydrophobic tail of the lipid bilayer. The non-polar-to-polar variant (p.Phe419Ser) alters the hydrophobicity in the membrane, compromising protein-lipid interactions. On the other hand, the Trp482 residue located in the extracellular linker region between transmembrane domains 5 and 6 is anchored to the membrane interfaces via its aromatic rings, mediating several molecular interactions that stabilize the structure of TMC1. This type of aromatic ring-based anchoring is also observed in homologous transmembrane proteins such as OSCA1.2. Conversely, the substitution of Trp with Arg (Trp482Arg) disrupts the cation-π interaction with phospholipids located in the outer leaflet of the phospholipid bilayer, destabilizing protein-lipid interactions. Additionally, Trp482Arg collapses the CH-π interaction between Trp482 and Pro511, possibly reducing the overall stability of the protein. In parallel with the molecular modeling, the two mutants degraded significantly faster compared to the wild-type protein, compromising protein stability.

**Conclusions:**

This results expand the genetic spectrum of disease-causing *TMC1* variants related to DFNA36 and provide insight into TMC1 transmembrane protein-lipid interactions.

**Supplementary Information:**

The online version contains supplementary material available at 10.1186/s12920-023-01766-7.

## Background

Hearing loss is the most common hereditary sensory disorder in human [1]. Approximately 50% of congenital hearing loss has a genetic cause [2]. In human, more than 150 genes have been reported to cause hearing loss (https://hereditaryhearingloss.org/) [3]. Determining the genetic cause of hearing loss enables timely and appropriate audiological rehabilitation. Furthermore, functional classification of deafness-related genes, including hair bundle development and functioning, have provided insight into genotype- and mechanism-based pharmacological approaches and gene therapy [4].

TMC1 is one of the member of TMC protein family, which includes eight members, TMC1-8. TMC1/2 are expressed in hair cells of the inner ear, whereas TMC4 is expressed in kidney, small intestine and colon, and TMC6/8 are expressed in keratinocytes [5, 6]. These expression patterns indicate the unique mechanosensory transmission properties of TMC1/2. *TMC1*, which encodes transmembrane channel-like protein 1, forms the mechanoelectrical transduction (MET) channel in auditory hair cells, which is necessary for auditory function [7]. The MET channel converts sound into electrical signals, enabling hearing [8]. TMC1 consists of 10 transmembrane domains, and transmembrane domains 4–7 form the ion-conducting pore of the TMC1 channel [9]. A human TMC1 homology model based on the cryo-EM structure of *C. elegans* TMC1 showed that the putative ion-conducting pore site comprised two basic residues and five acidic residues, which are essential for calcium ion permeability [10]. Also, TMC1 is an important regulator of membrane homeostasis induced by MET channels in hair cells [11]. TMC2 is also present in these cells, at least transiently during early developmental stages. However, the temporal expression of *TMC1* and *TMC2* is markedly different. *TMC2* expression in the cochlea abruptly decreases postnatally, creating an absolute requirement for *TMC1* at later stages [12]. By testing both single and double *TMC1* knockouts [5, 13–15], expression of *TMC2* is insufficient to fully restore their hair cell function.

*TMC1* disease-causing variants are known to result in both autosomal dominant (DFNA36) and autosomal recessive (DFNB7/11) non-syndromic hearing loss. Specifically, DFNA36 is characterized by post-natal progressive hearing loss with a predominance of high-frequency deterioration. In contrast, DFNB7/11 is associated with congenital severe-to-profound hearing loss. Importantly, neither DFNA36 nor DFNB7/11 has been observed to present vestibular symptoms. Of 125 pathogenic *TMC1* variants identified, only 8 are classified as autosomal dominant, which hampers the analysis of genotype-phenotype correlations for DFNA36 [16, 17]. Because *TMC1*-associated DFNA36 hearing loss is late onset and progressive, gene therapy could prevent disease progression [17]. The effect of gene therapy on *TMC1*-associated DFNA36 has been assessed by means of RNA interference and the CRISPR-Cas9 system, suggesting the possibility of clinical application [18, 19]. Given this, uncovering novel *TMC1* variants and evaluating their pathogenic mechanism is crucial.

In this study, we identified two novel *TMC1* variants underlying DFNA36 in transmembrane domain 4 (c.1256T > C:p.Phe419Ser) and the extracellular linker region between transmembrane domains 5 and 6 (c.1444T > C:p.Trp482Arg). Also, we predicted their pathogenicity based on 3D modeling, structural analysis and in vitro studies. The results expand the genetic spectrum of disease-causing *TMC1* variants underlying DFNA36 and further provide structural impact of these variants in context of the TMC1 transmembrane protein-lipid interactions.

## Methods

### Participants

This study was a retrospective review using the in-house databases of genetic hearing loss in Seoul National University Hospital. Two unrelated Korean families with causative *TMC1* variants responsible for hearing loss, segregating as a dominant trait, were included in the analysis. All procedures in this study were approved by the Institutional Review Board of Seoul National University Hospital (IRB-H-0905-041-281). Written informed consent was obtained from all participants, or from the legal guardians of pediatric participants.

### Molecular genetic testing

Genomic DNA was extracted from peripheral blood using the standard procedure and was subjected to exome sequencing using a Sure Select 50 Mb Hybridization and Capture Kit and a HiSeq2000 platform in four proband samples. The paired-end read length was 100 bp, and the reads were aligned using the University of California Santa Cruz (UCSC) hg19 reference genome browser (https://genome.ucsc.edu/). As described previously [20–24], bioinformatics analysis and strict filtering were performed to retrieve candidate variants of the autosomal-dominant genes responsible for NSHL as follows: (i) non-synonymous single nucleotide polymorphisms (SNPs) with quality scores > 30 and read depths > 20 were selected. (ii) variants with minor allele frequencies (MAFs) ≤ 0.001 were chosen based on their entries in several databases, including the Genome Aggregation Database (gnomAD, https://gnomad.broadinstitute.org/). (iii) filtering was performed based on known deafness genes. (iv) variants with MAFs ≤ 0.001 were analyzed in ethnically matched controls (Korean Reference Genome Database (KRGDB), http://152.99.75.168:9090/KRGDB/welcome.jsp) consisting of 1722 Korean individuals (3444 alleles). (v) the pathogenic potential of each variant was determined using in silico tools (Combined Annotation Dependent Depletion (CADD), https://cadd.gs.washington.edu/ and Rare Exome Variant Ensemble Learner (REVEL), https://sites.google.com/site/revelgenomics/). In addition, we used the GERP + + score from the UCSC Genome Browser (http://genome.ucsc.edu/) to estimate the evolutionary conservation of the amino acid sequences. Further, compatibility with inheritance patterns and audiological phenotypes was evaluated. (vi) The candidate variants were confirmed by Sanger sequencing, and a segregation study was performed using parental DNA samples. The pathogenicity of the two novel *TMC1* variants were classified using the American College of Medical Genetics and Genomics/Association for Molecular Pathology (ACMG/AMP) guidelines for genetic hearing loss [25]. The number of candidate variants of the two unrelated Korean families is presented (Additional file 1: Fig. S1).

### Clinical phenotyping

Clinical phenotyping, including audio-vestibular assessments and radiological evaluations, were performed. The hearing thresholds for six different octaves (0.25, 0.5, 1, 2, 4, and 8 kHz) were evaluated by pure-tone audiometry (PTA). The mean hearing threshold was calculated as the average of the thresholds at 0.5, 1, 2, and 4 kHz, and severity was classified as mild (20–40 dB), moderate (41–55 dB), moderately severe (56–70 dB), severe (71–90 dB), or profound (> 90 dB). In audiograms, the hearing-loss configuration was classified as down-sloping, rising, or flat. The down-sloping configuration was defined as a difference in thresholds between 0.25 and 8 kHz of > 20 dB HL and the threshold met the increase from 0.25 to 8 kHz.

### Structural modeling

In this study, two structural models of human TMC1 were generated using the SWISS-MODEL automated protein structure modeling engine and the Alphafold Protein Structure Database [26, 27]. The first model was constructed using the Cryo-EM structure of *C. elegans* TMC1 (PDB ID: 7usw) as a template and the SWISS-MODEL engine was used to analyze the structural impact of the p.Phe419Ser variant. The second model was generated using the Alphafold structure model of human TMC1 to analyze the p.Trp482Arg mutant, as the Trp482 residue is located in an intrinsically disordered region whose structure cannot be determined by Cryo-EM. The lipid bilayer-embedded protein database (http://memprotmd.bioch.ox.ac.uk/home/) was used to construct membrane embedded models of TMC1 [28]. The effect of *TMC1* missense variants on intramolecular interactions such as hydrophobic interaction, CH-π interaction, aromatic π-stacking was evaluated with PyMOL software (v. 2.4.1; PyMOL Molecular Graphics System v. 2.0, Schrödinger Inc., New York, NY, USA, https://pymol.org/2/), DynaMut server (http://biosig.unimelb.edu.au/dynamut/), DynaMut2 server (https://biosig.lab.uq.edu.au/dynamut2/), and mCSM membrane server (https://biosig.lab.uq.edu.au/mcsm_membrane/) to predict the effects of *TMC1* variants on structural stability. Figures were created using PyMOL software.

### Western blot

The HEK293T cells were cultured in DMEM at 37℃ and humidified air of a 5% CO2 incubator. The cells were transiently transfected with pCMV6-TMC1 wild type-myc-DDK, pCMV6-TMC1 F419S-myc-DDK and pCMV6-TMC1 W482R-myc-DDK, expressing plasmids using Lipofectamine 3000 (L3000015, Invitrogen) according to the manufacturer’s instructions. Whole proteins were separated using 10% sodium dodecyl sulfate-polyacrylamide gel electrophoresis (SDS-PAGE) and transferred to 0.45 μm polyvinylidene difluoride (PVDF) membranes. The membranes were incubated with 5% skim milk to block nonspecific binding at room temperature for 1 h. Membrane blots were incubated against Flag-tag antibody (F3165, Sigma) and β-actin antibody (A1978, Sigma). The membranes with bound primary antibodies were incubated with anti-mouse secondary antibodies that were conjugated horseradish peroxidase (HRP) for 1 h at room temperature. The protein band was detected using X-ray films. β-actin levels were used as loading controls. The intensity of bands was measured using the Image J software.

## Results

### Clinical profiles

**Fig. 1:**
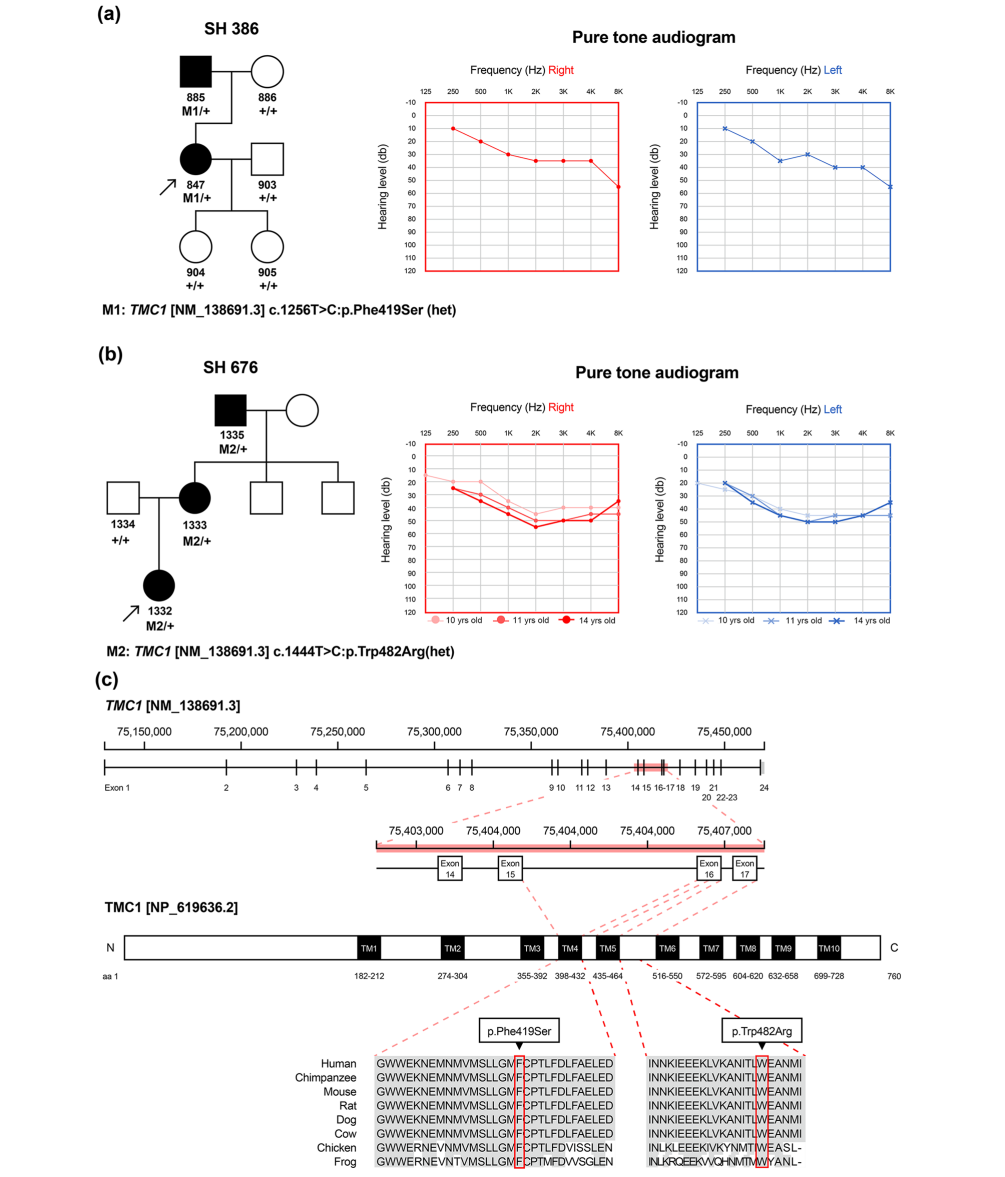
(**a, b**) Pedigrees of the two families, segregation of the respective *TMC1* variants, and the audiological phenotypes of the probands. Bilateral moderate sensorineural hearing loss was evident, exhibiting a down-sloping configuration, in the audiograms of the two probands. Progressivity of hearing loss was noted in serial audiograms of SH676-1332 as hearing declines with age. (**c**) Physical map and conserved residues of TMC1, which consists of 10 transmembrane domains. The domain structure of TMC1 was constructed based on the Universal Protein Resource (UniProt) database. The two variants c.1256T > C:p.Phe419Ser in SH386 and c.1444T > C:p.Trp482Arg in SH676 are located in transmembrane domain 4 and the extracellular linker region between transmembrane domains 5 and 6, respectively. Conservation of the affected residues (Phe419 and Trp482) among species was documented for the two *TMC1* variants identified in this study

In the SH386 family, age of onset for hearing loss and age of ascertainment for the proband (SH386-847) were the early 30s and late 30s, respectively. The audiogram showed bilateral moderate sensorineural hearing loss (SNHL) with a down-sloping configuration. Temporal bone computed tomography (CT) showed no inner ear anomaly. A review of the medical history and physical examination of the proband did not show other symptoms or underlying disease, only tinnitus without vertigo or dizziness. The father (affected individual) developed hearing loss without a vestibular phenotype (Fig. 1a). In the SH676 family, the proband SH676-1332 (early teens at ascertainment) manifested bilateral moderate SNHL with a down-sloping configuration. The proband’s hearing (SH676-1332) deteriorates with age. Serial audiograms (over the 4 years observation) of the proband SH676-1332 revealed a mild progression of hearing loss. Bilateral hearing aids were fitted when the proband was 12 years old. Neither inner ear anomalies nor brain lesions were detected by temporal bone CT and brain magnetic resonance imaging (MRI). The mother and grandfather (affected individuals) experienced progressive hearing loss in their early 30s. The pedigrees of the two unrelated families indicated autosomal dominant inheritance of hearing loss (Fig. 1a, b). Neither family reported subjective dizziness or vertigo, and no vestibular function deficits were observed based on the video head impulse test.

### Genotyping of novel TMC1 variants

By exome sequencing, we identified two novel *TMC1* variants underlying DFNA36 in transmembrane domain 4 (c.1256T > C:p.Phe419Ser) and the extracellular linker region between transmembrane domains 5 and 6 (c.1444T > C:p.Trp482Arg) (Fig. 1c). The two variants were extremely rare in the KRGDB (1722 individuals) and Global Minor Allele Frequency database, which met the standard for moderate evidence (PM2) in the ACMG/AMP guidelines on hearing loss. CADD and REVEL in silico analyses of p.Phe419Ser yielded high scores of 25.8 and 0.832, respectively, satisfying the criteria for supporting evidence (PP3) in the ACMG/AMP guidelines on hearing loss. Also, Phe419 is highly conserved among TMC1 orthologs of several species, with a high GERP + + score of 6.02. CADD and REVEL in silico analyses of p.Trp482Arg yielded scores of 28 and 0.701, respectively, satisfying the criteria for supporting evidence (PP3). Phe419 is highly conserved among TMC1 orthologs of several species, with a high GERP + + score of 6.02. In the SH676 family, supporting evidence (PP1) was further assigned as the segregation trait was confirmed as autosomal dominant in the two affected relatives. Accordingly, p.Phe419Ser and p.Trp482Arg were classified as variants of uncertain significance (VUS) based on the ACMG/AMP guidelines on hearing loss (Table 1).

**Table 1:** *TMC1* novel dominant variants and their pathogenicity prediction analysis

Proband	Genomic Position: Change (GRCh37/hg19)	HGVS		Location (Exon/Domain)	Zygosity /Inheritance	*In silico* Predictions		Alternative Allele Frequency		ACMG/AMP 2018 Guideline		Clinvar
		Nucleotide change	Amino Acid change			CADD Phred	REVEL	KRGDB (1722 individuals)	GMAF (gnomAD)	Criteria	Classification	Classification
SH 386–847	Chr9:75406833T-C	c.1256T > C	p.Phe419Ser	Exon 16/TM4	Het/AD	25.8	0.832	Absent	Exome (0.00001194) Genome (Absent)	PM2, PP3	VUS	ND
SH 676–1332	Chr9:75407146T-C	c.1444T > C	p.Trp482Arg	Exon 17/Linker between TM5/6	Het/AD	28	0.701	0.000293945	Exome (0.000003977) Genome (Absent)	PM2, PP1, PP3	VUS	ND

^*^ Het, heterozygote; AD, Autosomal dominant; TM, Transmembrane domain; MAF, minor allele frequency; VUS, variant uncertain significance; ND, not determined

Refseq transcript accession number NM_138691.3; Refseq protein accession number NP_619636.2

HGVS: Human Genome Variation Society (https://www.hgvs.org/); Sequence Variant Nomenclature (http://varnomen.hgvs.org/); CADD: Combined Annotation Dependent Depletion (https://cadd.gs.washington.edu/); REVEL: Rare Exome Variant Ensemble Learner (https://sites.google.com/site/revelgenomics/); KRGDB: Korean Reference Genome Database (http://coda.nih.go.kr/coda/KRGDB/index.jsp); gnomAD: The Genome Aggregation Database (https://gnomad.broadinstitute.org/); ACMG/AMP 2018 guideline (http://wintervar.wglab.org/); Clinvar (https://www.ncbi.nlm.nih.gov/clinvar/)

There are two primary NGS-based copy number variation (CNV) detection algorithms: read depth and paired-end mapping. These employ statistical models and clustering approaches, respectively, for CNV detection. As delineated in a previous study [29], we applied the CNV detection method known as consistent count region (CCR)–CNV to determine the presence or absence of CNVs in the patient’s exome for nonsyndromic deafness genes (https://hereditaryhearingloss.org/), specifically identifying for a double hit. The optimal thresholds for heterozygous deletion and duplication were established at 0.744 and 1.273, respectively [29]. In CCR-CNV analysis, neither deletions nor duplications were observed in the exomes of the two probands. More specifically, we visually inspected all *TMC1* exons and confirmed the absence of CNVs using the Integrative Genomics Viewer (IGV) (Additional file 2: Fig. S2).

### Structural analysis of TMC1 variants

**Fig. 2:**
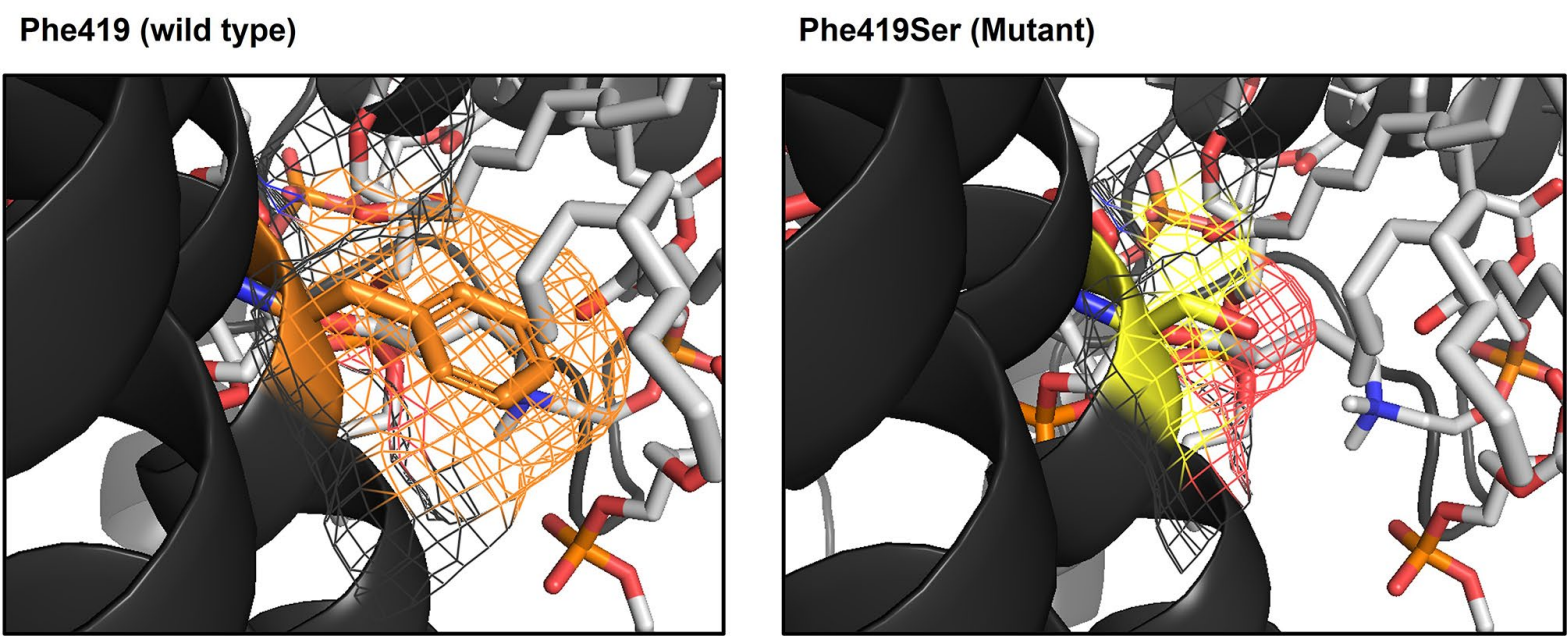
Three-dimensional modeling and structural analysis of p.Phe419Ser *TMC1* variants. Three-dimensional model structure of TMC1 wild type and p.Phe419Ser mutant. Transmembrane domain 4 (black), Phe419 residue (orange), p.Phe419Ser mutagenesis (yellow), and stick models of phosphatidylcholine (gray). Compromised protein-lipid interaction of p.Phe419Ser mutant in membranous condition

Transmembrane domains 4–7 form the ion conduction pathway, which is essential for hearing [9]. Phe419 in transmembrane domain 4 is facing outward from the ion conduction pore of the TMC1 channel, which is buried in hydrophobic tails of lipid bilayer. Therefore, non-polar-to-polar variants (p.Phe419Ser) of *TMC1* in which hydrophobic Phe is substituted for hydrophilic Ser have altered hydrophobicity, compromising protein-lipid interactions (Fig. 2). The protein stability/pathogenicity prediction servers, mCSM-membrane, DynaMut, and DynaMut2, consistently predicted a negative effect of the missense variant (p.Phe419Ser) on protein stability (Additional file 3: Table S1), supporting detrimental effect of p.Phe419Ser although stability prediction cannot fully reflect protein-lipid interaction [30–32].

**Fig. 3:**
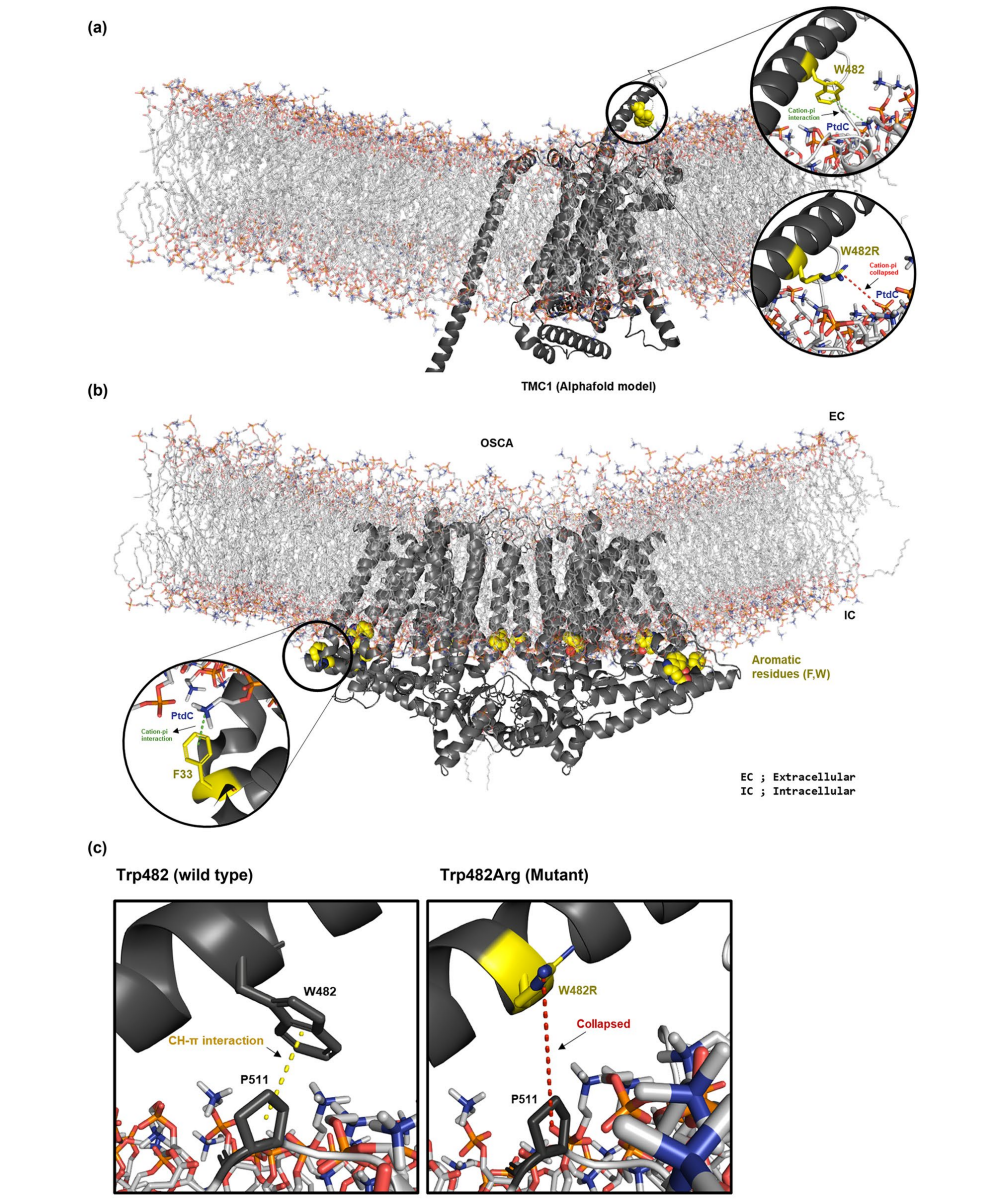
Three-dimensional modeling and structural analysis of p.Trp482Arg *TMC1* variants. (**a**). Three-dimensional model of TMC1 (black) wild type and p.Trp482Arg mutant. (Right-upper circle, wild type) Cation-π interaction between Trp482 (yellow) and phosphatidylcholine. (Right-lower circle, p.Trp482Arg mutant) Collapsed cation-π interaction due to substitution of Trp with Arg (yellow). (**b**) Aromatic ring-based anchoring, showing the cation-π interaction around the lipid-bilayer edge in the OSCA1.2. (**c**) Intra-protein interactions of Trp482 (black). (Left, wild type) CH-π interaction between Trp482 and Pro511. (Right, p.Trp482Arg mutant) Collapsed CH-π interaction due to substitution of Trp with Arg (yellow)

Trp482 in the extracellular linker region between transmembrane domains 5 and 6 is anchored via aromatic rings to membrane interfaces (Fig. 3a). Such aromatic ring-based anchoring is also observed in homologous proteins such as OSCA 1.2 (Fig. 3b) and TMEM 16 [5, 6]. Conversely, substitution of Trp with Arg (p.Trp482Arg) collapsed the cation-π interaction with phospholipids (e.g., the positively charged head of phosphatidylcholine) around the lipid-bilayer edge (Fig. 3a), disrupting protein-lipid interactions. Moreover, p.Trp482Arg collapsed CH-π interaction between Trp482 and Pro511 (Fig. 3c), possibly reducing protein stability. Also, *in silico* analyses consistently predicted a negative effect of the missense variant (p.Trp482Arg) on protein stability (Additional file 3: Table S1).

### Protein stability of TMC1 mutants

**Fig. 4:**
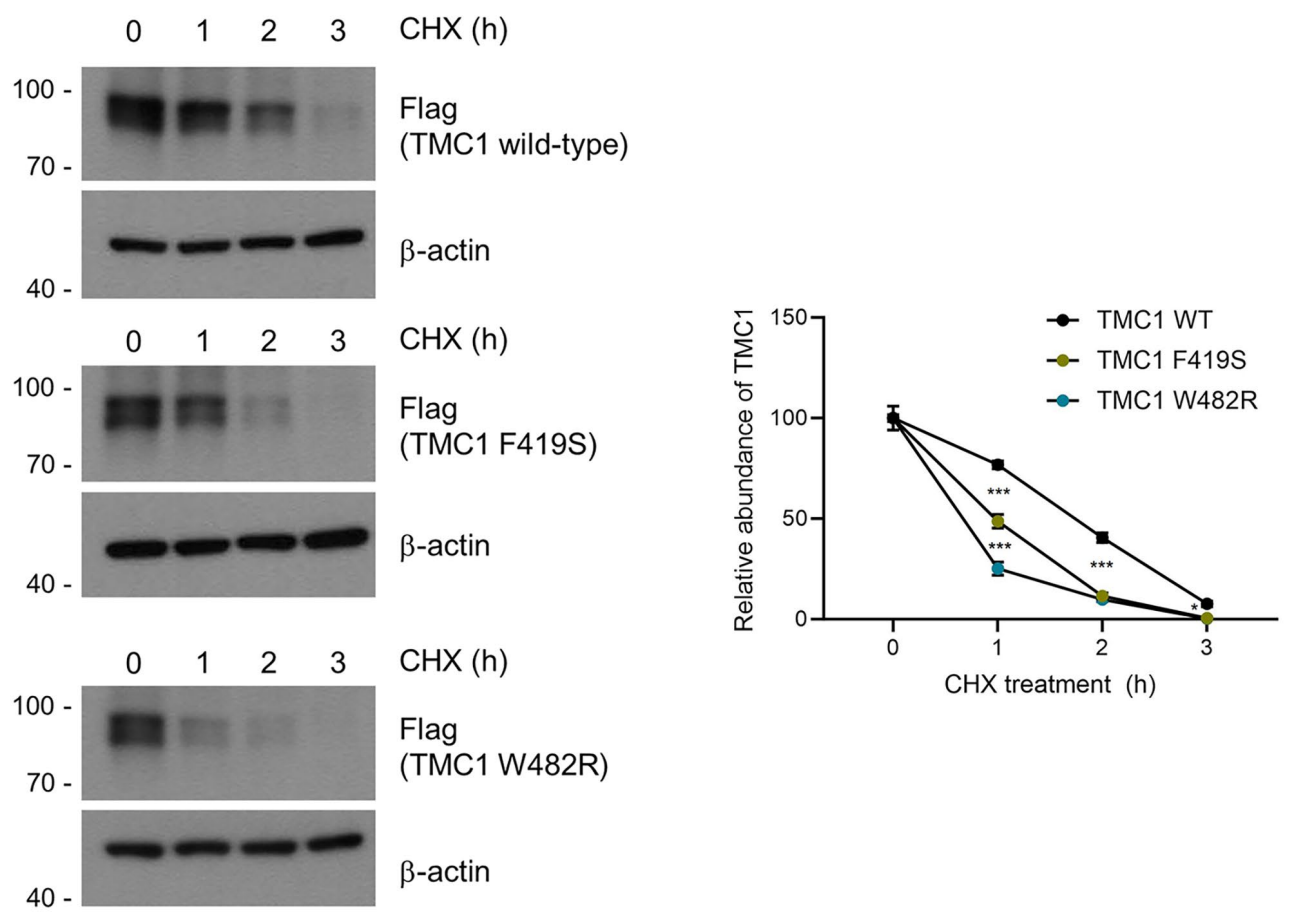
Comparative analysis of the stability between TMC1 wild-type, p.Phe419Ser and p.Trp482Arg proteins using CHX chase assays in a transient overexpression system. HEK293T cells overexpressing TMC1 were treated with CHX at a concentration of 80 µg/ml for a duration of up to 3 h to inhibit general translation. In the immunoblots, the observed two bands for TMC1 wild-type and mutants might represent phosphorylated TMC1 [9]. The CHX chase assay was conducted once, with three measurements acquired throughout the experiment. Consequently, each experimental condition had a sample size of three. The original blots were represented in Additional file 4: Fig. S3

The western blot analysis showed that both the wild-type and the two mutant proteins, carrying missense variants (p.Trp482Arg and p.Phe419Ser), were expressed as the expected molecular weight (87.8 kDa) from the tagged TMC1 mutants (Fig. 4). In comparison to the wild-type protein, steady-state expression level of the two mutant proteins did not differ (Fig. 4). To investigate whether the two variants contribute to destabilize the TMC1 protein, we conducted cycloheximide (CHX) chase assays that inhibit protein synthesis. HEK293T cells were transfected with the wild-type and the two mutant plasmids for 24 h, and then treated with CHX (80 µg/ml) for 1, 2, and 3 h, respectively. The results showed that, at all measured time points, the two mutants degraded significantly faster compared to the wild-type protein. In summary, the two *TMC1* variants (p.Trp482Arg and p.Phe419Ser) could alter lipid-protein interactions, thereby compromising protein stability. The original blots were represented in (Additional file 4: Fig. S3).

## Discussion

In this study, we identified two novel *TMC1* variants related to DFNA36 in transmembrane domain 4 (p.Phe419Ser) and the extracellular linker region between transmembrane domains 5 and 6 (p.Trp482Arg), and predicted the pathogenicity of those variants by 3D modeling and structural analysis. Notably, the two mutant proteins encoded by *TMC1* missense variants disrupted protein-lipid interactions in membranes, compromising protein stability. The results expand the genetic spectrum of disease-causing *TMC1* variants related to DFNA36 and offer insight into TMC1 transmembrane protein-lipid interactions.

Transmembrane domains 4–7 form the ion conduction pathway of TMC1 channels, which is essential for auditory function [9]. Phe419 in transmembrane domain 4 is adjacent to Gly417, Met418, and Thr422, which are crucial residues for the ion conduction pore of TMC1 channels. Substitution of cationic amino acids, including Arg and Lys, in transmembrane domain 4 (Gly417, Met418, and Thr422) alters the properties of the ion conductance pore, leading to decreased calcium ion permeability due to cation-cation repulsion [33]. Specifically, p.Met412Lys in Beethoven mouse, which is homologous to p.Met418Lys, not only changes ion channel properties at the molecular level but also causes progressive hearing loss analogous to DFNA36 in human [34]. Likewise, p.Thr416Lys in mouse, which is homologous to p.Thr422Lys, causes loss of outer hair cells during development, resulting in progressive hearing loss analogous to DFNA36 in human. The results suggest that transmembrane domain 4 is crucial for the function of TMC1, giving rise to hearing loss when mutated. However, structural modeling showed that Phe419 in transmembrane domain 4 faces outward toward the pore of the TMC1 channel, facing hydrophobic tail of lipid bilayer, is merit attention. As a result, rather than directly affecting ion permeability, p.Phe419Ser likely compromised the TMC1 protein-lipid interaction in the membrane, ultimately leading to progressive, high-frequency dominant hearing loss consistent with that caused by DFNA36. Because Ser is hydrophilic, changes in hydrophobicity due to substitution of Phe for Ser may destabilize TMC1 in the lipid bilayer. As for other membrane proteins such as CFTR (cystic fibrosis transmembrane conductance regulator) and ATP7B (copper-transporting ATPase 2), non-polar-to-polar missense variants in transmembrane domains destabilize the structure, leading to diseases such as cystic fibrosis and Wilson’s disease [35]. p.Trp77Ser (non-polar-to-polar variant) in transmembrane domain 2 of the membrane protein Connexin 30.2/Connexin 31.4 (CX30.2/CX31.3) causes hearing loss, possibly by introducing structural instability and promoting mutant CX30.2/CX31.3 aggregation in the endoplasmic reticulum [36].

It is of particular interest to note that the Phe419 residue is highly conserved in the homologous protein OSCA1.2. The Phe residue is conserved at position 440 in OSCA1.2, corresponding to position 419 in TMC1. Also, it is known that the TM4 sequence of TMC1 including Phe419 is almost completely conserved from mammals to amphibians [37] indicating the importance of Phe419 in maintaining the structural stability of TMC1 and its homologous proteins. Although *C. elegans* TMC1 has a Val residue in place of Phe, it is still notable that both amino acids are hydrophobic, suggesting that maintaining hydrophobicity at this position is crucial for the stability of TMC1.

The functional roles of aromatic residues at the membrane interfaces are well demonstrated by several studies [38–40]. Importantly, Trp is preferentially distributed at the lipid-water interface, stabilizes membrane protein localization. It was reported that the cation-π interaction between aromatic residue and cationic head of phospholipids is one of the major sources of those anchoring process [41]. As evidenced by 3D modeling, Trp482 locates at the membrane interface between transmembrane domains 5 and 6 and mediates several molecular interactions which stabilize membrane embedded TMC1 structure. Such aromatic ring-based anchoring is commonly observed in channel proteins including TMC1 homologous proteins, such as OSCA1.2 (Fig. 3b) and TMEM16 [42]. Substitution of Trp482 for positively charged Arg destroyed the cation-π interaction with phospholipids (e.g., the positively charged head of phosphatidylcholine) around the lipid-bilayer edge, resulting in misfolding and destabilization of TMC1. Thus, p.Trp482Arg likely compromises protein-lipid interactions to destabilize TMC1, which might be linked with hearing loss. Upon the Alphafold model, p.Trp482Arg collapses putative CH-π interaction between Trp482 and Pro511, substantially reducing protein stability. However, given that both Trp482 and Pro511 are located within an unidentified region, even in the CryoEM study [10], further validation is necessary to establish structural instability resulting from deficiencies in those molecular interactions. Alternatively, p.Trp482Arg (i.e., neutral-to-positively charged missense variant) may perturb ion permeability due to cation-cation repulsion between calcium ion and the positively charged Arg residue, although Trp482 is far from the ion conduction pore of the TMC1 channel. This hypothesis is supported by the observation that the introduction of cationic residues around TMC1 ion pore significantly decreases the ion transduction and selectivity of TMC1 [9].

The structural stability of TMC1 may be linked to intra-protein interactions, protein- protein interactions, and/or the properties of ion conductance pores. p.Ser320Arg, which is causative of DFNA36, may involve the pathogenic mechanism of destabilization due to compromised intra-protein interactions [43]. p.Asp572Asn, which is related to DFNA36, is linked to dysfunction due to derangement of protein-protein interactions, hindering the interaction of TMC1 and LHFPL5 [44]. Also, p.Gly417Arg, p.Met418Lys and p.Thr422Lys are attributable to the pathogenic mechanism of dysfunction by altering the net charge of ion conductance pores [33]. Our results suggest that, in addition to compromised intra-protein interactions, TMC1 p.Phe419Ser and p.Trp482Arg may disrupt protein-lipid interactions, providing new insight into the pathogenic mechanism of DFNA36. This is in line with a recent report that TMC1-related hearing loss may involve alterations in membrane homeostasis [11]. However, it was challenging to confirm these structural predictions through in vitro functional assays, due to the insufficient membrane trafficking of human TMC1 in over-expression system. This is also supported by the fact that the most of TMC1 functional studies have been performed using in vivo models including KO mouse and *C. elegans*, instead of in vitro cell based functional assays [45–47]. Indeed, our preliminary experiments showed that a majority of the over-expressed TMC1 proteins were aggregated in cytosolic spaces (Data not shown). This may suggest that the identification of accessory proteins, such as TMIE or CIB2, are necessary for the proper folding or membrane trafficking of human TMC1. This is further supported by the fact that TMC1 isolated from *C. elegans*, the structure of which was recently determined through cryo-EM, exists as a complex rather than in apo structures (10).

The difference in hearing level at 8 kHz between SH676 and SH386 is noteworthy and raises questions about the compensation mechanism of TMC2. TMC2 is only transiently expressed during the developmental stage of the rodent cochlea (P2-P10), while TMC1 is constitutively expressed by P7 [13]. The distinct temporal expression of TMC1/2 may preclude the possibility of the compensation mechanism. Additionally, TMC2 expression has been found to be insufficient to fully restore cochlea function in *TMC1* knock-out mice [14, 15]. TMC2 is unable to interact with CIB2, one of the core proteins of MET channel complex, due to the difference in N-terminal sequence [15], suggesting TMC2 cannot form the MET channel complex without TMC1. Furthermore, TMC2 is expressed at the apex of the cochlea, whereas TMC1 is expressed at the base of the cochlea [48]. The distinct tonotopic expression of TMC1/2 also suggests that TMC2 is less likely to compensate for the loss of TMC1 function in the high-frequency region. Nonetheless, as the cochlear development period of mice and humans are different, the expression of TMC2 in teens (SH676) compared to 30s (SH386) may partially compensate for the loss of TMC1 function, which would require elucidation of the temporal expression of TMC1/2 in the human cochlea. Moreover, the functional epistasis between MET channel complexes, such as procadherin-15 and TMHS/LHFPL5, may contribute to the difference in auditory phenotype (i.e., hearing level at 8 kHz) between the two *TMC1* mutants.

Based on *in silico* protein stability predictions (Additional file 3: Table S1) coupled with protein stability assay using CHX, the Trp482Arg variant appears to destabilize TMC1 protein more significantly than the Phe419Ser variant. This pronounced destabilization might explain the faster progression of hearing loss observed in SH 676 family segregating with the Trp482Arg variant. Alternatively, the varying onset of hearing loss between the two families could be attributed to the variable expressivity of *TMC1* variants [49]. In addition, differences in the epigenome and environmental factors might also influence the variable expressivity, potentially affecting the age-of-onset for hearing loss. In line with this, a previous study has reported that hearing deterioration in DFNA36 patients typically begins in their 1st or 2nd decade, which is consistent with our findings [17].

*TMC1* variants are causative of autosomal dominant (DFNA36) and autosomal recessive (DFNB7/11) non-syndromic hearing loss. However, in the literature, most of the *TMC1* variants are responsible for DFNB7/11, whereas only eight variants (p.Ser320Arg, p.Tyr381Asn, p.Gly417Arg, p.Met418Lys, p.Asp543Asn, p.Asp572Asn, p.Asp572His, p.Thr422Lys) are reported as causative for DFNA36 [16, 33, 43, 50–56]. In this study, two novel *TMC1* variants (p.Phe419Ser and p.Trp482Arg) related to DFNA36 cause high-frequency, late-onset, progressive hearing loss. Vestibular symptoms, including vertigo, were absent in the affected individuals. The clinical phenotypes are consistent with previously reported dominantly inherited heterozygous *TMC1* variants, although the severity of hearing loss differed between variants (Table 2). Theoretically, much genotype information on the deafness gene and audiological data should be necessary to predict the clinical course and genotype-phenotype correlation, and for timely and appropriate audiological rehabilitation of genetic hearing loss. Given this, further reports of families segregating *TMC1* dominant variants are needed to evaluate the clinical phenotypes caused by *TMC1* variants, thereby enabling genotype-phenotype correlations.

**Table 2:** Clinical phenotypes of the novel *TMC1* dominant variants and literature review

Variant		Inheritance pattern	Ethnicity	Severity	Configuration	Progression	Tinnitus	Vertigo	Reference
Nucleotide change	Amino acid change								
c.1256T > C	p.Phe419Ser	AD	Korean	Moderate	High-frequency SNHL (down-sloping)	NA	Yes	No	This study
c.1444T > C	p.Trp482Arg	AD	Korean	Moderate	High-frequency SNHL (down-sloping)	Yes	No	No	This study
c.960 C > G	p.Ser320Arg	AD	Polish	Mild	NA	NA	NA	NA	Mohamed Ahamed Hassan et al. 2015
c.1141T > A	p.Tyr381Asn	AD	NA	Moderately severe	High-frequency SNHL (down-sloping)	NA	NA	NA	Tina Likar et al. 2018
c.1249G > A	p.Gly417Arg	AD	Iranian	Profound	High-frequency SNHL (down-sloping)	Yes	NA	NA	Tao Yang et al. 2010
c.1253T > A	p.Met418Lys	AD	Chinese	Severe	High-frequency SNHL (down-sloping)	Yes	Yes	NA	Yali Zhao et al. 2014
c.1265 C > A	p.Thr422Lys	AD	NA	Profound	High-frequency SNHL (down-sloping)	Yes	NA	No	Maryline Beurg et al. 2021
c.1627G > A	p.Asp543Asn	AD	Japanese	Severe	High-frequency SNHL (down-sloping)	Yes	Yes	No	Shin-ya Nishio & Shin-ichi Usami 2022
c.1714G > A	p.Asp572Asn	AD	Japanese	Moderate	High-frequency SNHL (down-sloping)	NA	NA	NA	Shin-ya Nishio & Shin-ichi Usami 2022
c.1714G > C	p.Asp572His	AD	Caucasian	Profound	High-frequency SNHL (down-sloping)	Yes	NA	No	Kitajiri et al. 2007

* AD, autosomal dominant; SNHL, sensorineural hearing loss; NA, not available

## Conclusions

This results expand the genetic spectrum of disease-causing *TMC1* variants related to DFNA36 and provide insight into TMC1 transmembrane protein-lipid interactions.

### Electronic supplementary material

Below is the link to the electronic supplementary material.

Supplementary Material 1

Supplementary Material 2

Supplementary Material 3

Supplementary Material 4

## Data Availability

The datasets generated and analysed during the current study are available in the clinVAR repository, SCV002822952 (https://www.ncbi.nlm.nih.gov/clinvar/variation/VCV001895419.1) and SCV002822953 (https://www.ncbi.nlm.nih.gov/clinvar/variation/VCV001895420.1). All other relevant data of this study are available within the article and its Supplementary Material. Individual-level whole-exome sequence data has not been made publicly available for ethical reasons. However, they are available from the corresponding author on reasonable request.

## Abbreviations

TMC1: Transmembrane channel-like protein 1
MET: Mechanoelectrical transduction
DFNA: Deafness, Autosomal Dominant
OSCA: Hyperosmolality-gated calcium permeable channel
TMEM: Transmembrane protein
Cryo-EM: Cryogenic electron microscopy
CRISPR: Clustered regularly interspaced short palindromic repeats
Cas9: CRISPR-associated protein 9
PTA: Pure tone audiometry
SNHL: Sensorineural hearing loss
CT: Computed tomography
MRI: Magnetic resonance imaging
KRGDB: Korean reference genome database
ACMG: American college of medical genetics and genomics
AMP: Association for molecular pathology
CADD: Combined annotation dependent depletion
REVEL: Rare exome brainat ensmble learner
GERP: Genomic evolutionary rate profiling
VUS: Variants of uncertain significance
GMAF: Global minor allele frequency
Het: Heterozygote
AD: Autosomal dominant
TM: Transmembrane domain
ND: Not determined
gnomAD: The genome aggreagation database
NA: Not available
CFTR: Cystic fibrosis transmembrane conductance regulator
ATP7B: Copper-transporting ATPase2
CX: Connexin
LHFPL: Lipoma HMGIC fusion partner
TMIE: Transmembrane inner ear
CALM1: Calmodulin 1
P: Postnatal day
CIB2: Ca^2+^ and integrin binding protein 2
