# Supplementary material for: Novel autosomal dominant TMC1 variants linked to hearing loss: insight into protein-lipid interactions
Source: BMC Med Genomics. 2023 Dec 8;16:320. doi: 10.1186/s12920-023-01766-7 (PMC10704677; doi:10.1186/s12920-023-01766-7)
Supplement: Supplementary file 1 — Supplementary Material 1 [file 12920_2023_1766_MOESM1_ESM.docx]

Supplementary Table 1. *in silico* prediction of stability and pathogenicity of TMC1 mutants

| ***TMC1* variants** | **mCSM-membrane** | | **DynaMut ΔΔG (kcal/moL)** | **DynaMut2 ΔΔG (kcal/moL)** |
| --- | --- | --- | --- | --- |
|  | **ΔΔG (kcal/moL)** | **Pathogenicity** |  |  |
| p.Phe419Ser | -0.517 (Destabilize) | Pathogenic | -0.726 (Destabilize) | -0.54 (Destabilize) |
| p.Trp482Arg | -0.544 (Destabilize) | Pathogenic | -1.22 (Destabilize) | -0.63 (Destabilize) |
