# Supplementary material for: Novel autosomal dominant TMC1 variants linked to hearing loss: insight into protein-lipid interactions
Source: BMC Med Genomics. 2023 Dec 8;16:320. doi: 10.1186/s12920-023-01766-7 (PMC10704677; doi:10.1186/s12920-023-01766-7)
Supplement: Supplementary file 2 — Supplementary Material 2 [file 12920_2023_1766_MOESM2_ESM.docx]

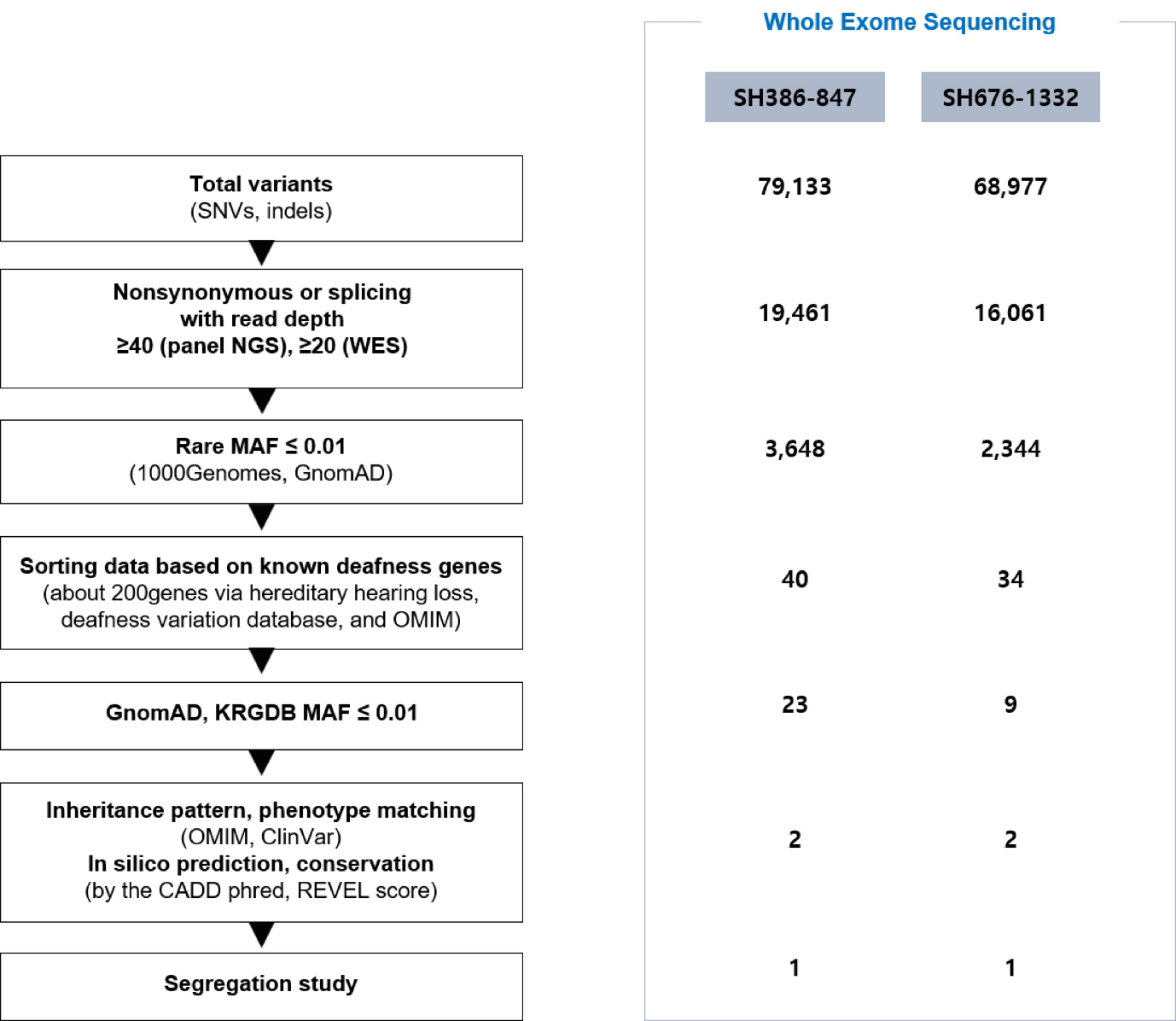


Supplementary Figure 1. Flow chart of bioinformatics analysis and strict filtering process of candidate variants linked to autosomal dominant NSHL. The number of candidate variants of two unrelated Korean families (SH386-847 and SH676-1332) is presented.
