# Supplementary material for: Novel autosomal dominant TMC1 variants linked to hearing loss: insight into protein-lipid interactions
Source: BMC Med Genomics. 2023 Dec 8;16:320. doi: 10.1186/s12920-023-01766-7 (PMC10704677; doi:10.1186/s12920-023-01766-7)
Supplement: Supplementary file 3 — Supplementary Material 3 [file 12920_2023_1766_MOESM3_ESM.docx]

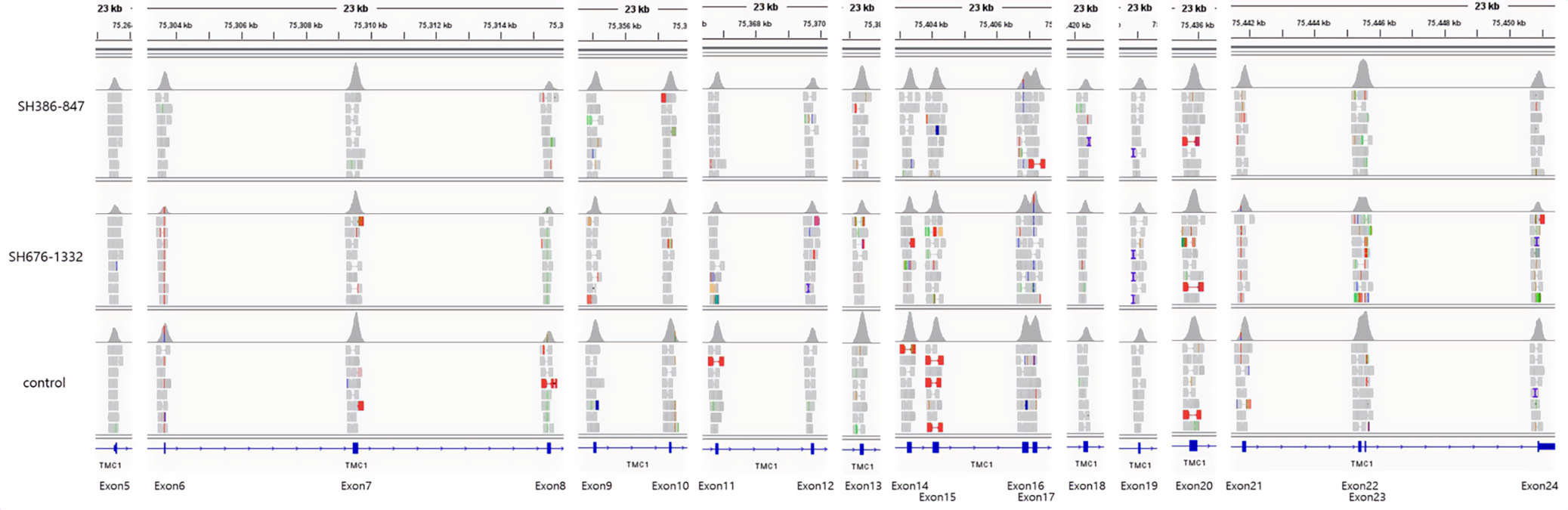


Supplementary Figure 2. Visualization of CCR-CNV analysis using Integrative Genomics Viewer (IGV). Neither deletions nor duplications were observed in the exomes of the two probands. All *TMC1* exons were visually inspected and we confirmed the absence of CNVs.
