# Supplementary material for: Novel autosomal dominant TMC1 variants linked to hearing loss: insight into protein-lipid interactions
Source: BMC Med Genomics. 2023 Dec 8;16:320. doi: 10.1186/s12920-023-01766-7 (PMC10704677; doi:10.1186/s12920-023-01766-7)
Supplement: Supplementary file 4 — Supplementary Material 4 [file 12920_2023_1766_MOESM4_ESM.docx]

**
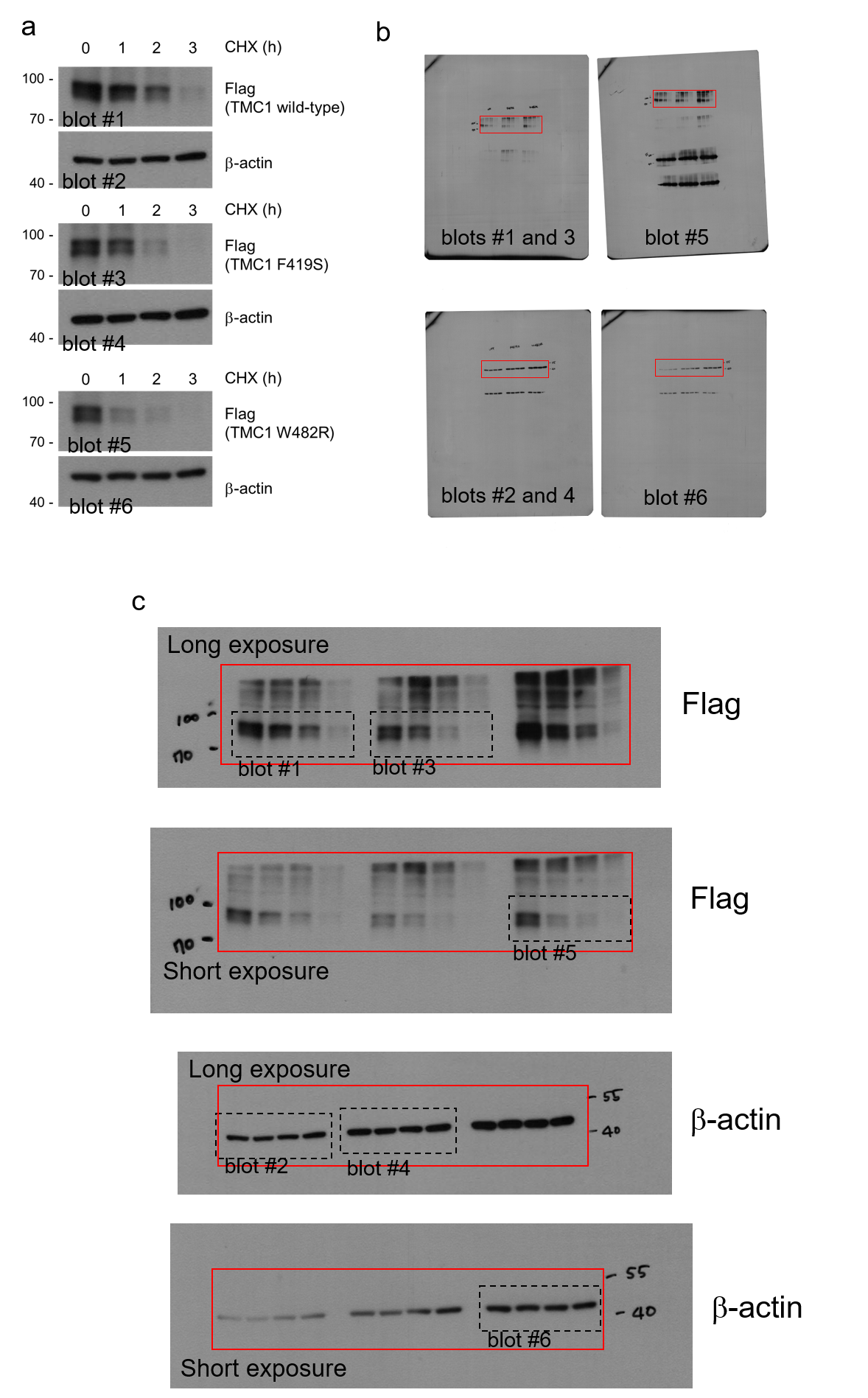
**

**Supplementary figure 3.** Original blots corresponding to Figure 4. (a) Figure 4 with specific blot numbering. (b) Full film displaying blots #1 to #6. Before antibody hybridization, blots were cut; the membrane edges are represented with red lines. Although membrane images were not captured, protein sizes from the marker proteins are indicated on the full film. (c) Enlarged views of the blots from the whole film. Similar to (b), red lines indicate the blot edges, while dotted lines represent the edges of cropped blots. Blots #1, #3, and #5 originate from the same membrane but are from images taken at different exposure times. Similarly, blots #2, #4, and #5 are from the same conditions.
